# Endovascular Treatment of Type A Aortic Dissection: A Systematic Review and Meta-Analysis Using Reconstructed Time-to-Event Data

**DOI:** 10.3390/jcm12227051

**Published:** 2023-11-12

**Authors:** Konstantinos S. Mylonas, Ioannis Zoupas, Panagiotis T. Tasoudis, Evangelos Vitkos, George T. Stavridis, Dimitrios V. Avgerinos

**Affiliations:** 1 Department of Cardiac Surgery, Onassis Cardiac Surgery Center, 176 74 Athens, Greece; ksmylonas@gmail.com (K.S.M.);; 2 School of Medicine, National and Kapodistrian University of Athens, Mikras Asias Str. 75, 115 27 Athens, Greece; 3 Surgery Working Group, Society of Junior Doctors, 151 23 Athens, Greece; tasoudis@gmail.com; 4 Department of General Surgery, General Hospital of Katerini, 601 00 Katerini, Greece

**Keywords:** TEVAR, type A aortic dissection, endovascular, TAAD

## Abstract

Objective: The undisputed gold standard of treatment for type A aortic dissections (TAAD) is open surgery. Anecdotal reports have assessed thoracic endovascular aortic repair (TEVAR) as a last resort for highly selected candidates. The present study aims to evaluate endovascular outcomes in TAAD patients who are unsuitable for open surgery whilst having TEVAR-compatible aortic anatomy. Methods: A PRISMA-compliant systematic search of the PubMed, Scopus, and Cochrane databases was performed up to 19 May 2022. Time-to-event data were reconstructed using Kaplan–Meier curves from the source literature. Results: In 20 eligible studies, 311 patients underwent TEVAR for acute, subacute, or chronic TAAD. Mean age at the time of the operation was 60.70 ± 8.00 years and 75.48% (95% Confidence Interval [CI], 60.33–88.46%) of the included patients were males. Mean operative time was 169.40 ± 30.70 min. Overall, 0.44% (95% CI, 0.00–4.83%) of the cases were converted to salvage open surgery. Technical failure, stroke, and endoleaks occurred in 0.22%, 0.1%, and 8.52% of the cohort, respectively. Thirty-day postoperative complication rate was 7.08% (95% CI, 1.52–14.97%), whereas late complications developed in 16.89% (95% CI, 7.75–27.88%) of the patients. One-, three-, and five-year survival rates were estimated at 87.15%, 82.52% and 82.31%, respectively. Reintervention was required in 8.38% of the cohort over a mean follow-up of 32.40 ± 24.40 months. Conclusions: TEVAR seems to be feasible in highly selected patients with TAAD who cannot tolerate open surgery. Overcoming technical limitations and acquiring long-term data are warranted to safely define the place of endovascular treatment in the armamentarium of TAAD repair.

## 1. Introduction

The spectrum of acute aortic syndrome (AAS) includes penetrating aortic ulcer, intramural hematoma, acute aortic dissection (AAD), and traumatic aortic injury [1]. AAD is the most frequent (80–90%) and severe form of AAS. Annually, it affects 6000–10,000 people in the United States [1,2] and approximately 6 per 100,000 people in the United Kingdom [3]. Stanford Type A Aortic Dissection (TAAD) involves any part of the aorta proximal to the origin of the left subclavian artery. It primarily affects males in the seventh decade of their lives and comprises about 2/3 of AADs [4,5,6]. TAAD has a mortality rate of 1–2% per hour and approximately half of these patients will expire within 24 h of disease onset [2,7].

The standard approach after TAAD diagnosis is direct transfer to the operating room for emergent open surgical repair [2]. Perioperative mortality has been estimated at 13–25%, while five- and ten-year survival rates have been reported to be around 70–75%, respectively [2,8]. Nevertheless, about one in four TAAD patients are deemed ineligible for surgery on account of severe comorbidities, advanced age, and/or patient refusal. Historically, this patient population has been managed medically with an in-hospital mortality of 60% [9,10]. Anecdotal reports have assessed thoracic endovascular aortic repair (TEVAR) as a last resort for poor surgical candidates [11]. Albeit promising, the most relevant published literature consists of small case series and no device has been formally approved for the endovascular repair of TAAD [10].

In the present study, we sought to systematically review and meta-analyze all available data exploring the role of endovascular treatment for acute, subacute, and chronic TAAD. To maximize the statistical robustness of our study, we reconstructed patient-level time-to-event data directly from the original Kaplan–Meier curves.

## 2. Methods

### 2.1. Systematic Search and Eligibility Criteria

Our study was conducted in accordance with the PRISMA statement (Preferred Reporting Items for Systematic Reviews and Meta-analyses) (Supplementary Figure S1) and was agreed upon by all authors [12]. Included studies were published in English and reported original patient data (clinical trials, cohort studies and case series). Only studies describing TEVAR for TAAD patients were considered. Reviews, meta-analyses, case reports, irrelevant studies, and manuscripts written in languages other than English were disqualified. Studies reporting on hybrid procedures were also excluded.

A systematic search was conducted up to 19 May 2022, and assessed studies from PubMed/MEDLINE, Scopus, and the Cochrane databases. Two independent reviewers (IZ and EV) searched these registries using the following algorithm: ((TEVAR) OR (ENDOVASCULAR) OR (Endograft)) AND ((“type A”) OR (“Debakey type I”) OR (“Debakey type II”)) AND (“aortic dissection”) NOT (“type B”). All conflicts regarding study eligibility were resolved with the assistance of a third, more experienced reviewer (PT). Our study protocol is registered at PROSPERO (ID number: CRD42022369078).

### 2.2. Data Extraction

Two independent reviewers (IZ, EV) performed data extraction using a standardized pre-designed formula for evidence collection. Conflicts were brought to a third researcher’s attention (PT) and resolved. The following patient features were extracted: age, sex, smoking habits, presence of diabetes, coronary artery disease (CAD), peripheral artery disease (PAD), chronic obstructive pulmonary disease (COPD), hypertension (HTN), connective tissue disease, dyslipidemia, prior cardiac surgery, prior aortic interventions, stroke history, aortic regurgitation or malperfusion prior to the operation, and heart and renal failure. Operation time (minutes), disease timeline (acute, subacute, or chronic TAAD), follow-up (months), vascular access site, type of device used, and concomitant procedures were also extracted. Finally, we reviewed information regarding the presence of endoleaks, development of new dissection (retrograde or not), technical failure, post-operative stroke, reintervention rates, conversion to salvage open surgery, as well as mortality rates (30-day and late (after 30 days)).

### 2.3. Statistical Analysis

#### 2.3.1. Data Pooling and Patient Feature Meta-Analysis

The techniques of Hozo et al. [13] and Wan et al. [14] were utilized to calculate the standard deviations (SDs) and mean values of continuous variables whenever medians and ranges or median and interquartile ranges were provided. To assess the occurrence rates of several events and the presence of between-study heterogeneity, we used the χ^2^-based Q statistic (significant if *p* < 0.1) and I^2^. All analyses were performed using STATA MP 16.0 (StataCorp LLC, College Station, TX, USA).

#### 2.3.2. Reconstruction of Patient Time-to-Event Data and Survival Meta-Analysis

We reconstructed individualized patient data (IPD) from Kaplan–Meier (KM) curves of the included studies, whenever they were available. We followed the methodology described by Wei et al. [15] and Guyot et al. [16]. High quality screenshots from the KM curves were acquired and Web Plot Digitizer was used to digitize them. Every added spot had to represent a specific Cartesian coordinate (also known as a time point and its corresponding survival information), so that this information could be extracted. Isotonic regression was used to identify deviations from monotonicity, which were managed with a pool-adjacent-violators algorithm. Extractable IPDs were then incorporated into STATA MP 16.0 in order to create a new KM curve that would include data from all available KM curves. This method provided us with pooled data regarding survival rates.

#### 2.3.3. Risk-of-Bias Assessment

The quality of the data presented in the included studies was evaluated using the National Heart, Lung, and Blood Institute (NHLBI) scale (Supplementary Table S1) [17]. The follow-up time sufficiency cut-off value was set at 12 months after the operation for each study.

## 3. Results

### 3.1. Literature Search

The initial search yielded a total of 1274 records. Ultimately, 20 studies met all of the inclusion criteria and were eventually included. Eight additional studies did not make it through the final selection process due to data overlap [18,19,20,21,22,23,24,25]. In total, we analyzed data from 311 patients with TAAD that underwent TEVAR (Table 1).

### 3.2. Basic Demographics and Medical History

Overall, 75.48% (95% CI, 60.33–88.46%) of the patients were males and mean age at the time of the operation was 60.70 ± 8.00 years. The vast majority of the patients had hypertension (90.97%; 95% CI, 81.60–97.79%). In terms of additional comorbidities, 49.97% (95% CI, 26.70–73.25%) were active smokers, 24.29% (95% CI, 6.97–45.87%) had dyslipidemia, 24.02% (95% CI, 7.77–43.94%) had a history of CAD, 20.18% (95% CI, 4.80–40.30%) had diabetes mellitus, and 20.03% (95% CI, 1.47–47.32%) had congestive heart failure (CHF).

Indications for TEVAR over open surgical repair were reported for 48.09% of our cohort. Age greater than 70 years was cited as the incentive for TEVAR in 19.01% (95% CI, 1.55–44.78%), previous cardiac surgery in 9.07% (95% CI, 0.13–24.94%), severe COPD in 5.54% (95% CI, 0.61–13.24%), a history of cerebrovascular accidents in 4.81% (95% CI, 0.00–16.12%), severe renal failure in 3.55% (95% CI, 0.02–10.56%), CHF in 3.54% (95% CI, 0.00–12.26%), American Society of Anesthesiologists (ASA) Class III or IV in 1.51% (95% CI, 0.00–10.42%), and patient preference in 1.06% (95% CI, 0.00–19.82%).

In addition, 11.49% (95% CI, 3.57–21.81%) had PAD, 7.42% (95% CI, 1.62–15.59%) had COPD, 7.79% (95% CI, 1.83–16.07%) had a history of cerebral stroke, and 7.20% (95% CI, 2.28–13.71%) suffered from renal failure. Only, two patients had connective tissue disorders. Lastly, 26.20% (95% CI, 11.42–43.43%) had undergone prior cardiothoracic surgical procedures, while 13.22% (95% CI, 3.29–26.51%) had prior aortic interventions performed. Specifically, seven (46.70%) patients had a history of aortic valve replacement, three (20.0%) were subjected to open aortic repair for dissection, three received a Bentall procedure, one (6.7%) a Wheat procedure and another (6.70%) patient had a history of a prior TEVAR. Significant cumulative details regarding patient demographics and medical history are provided in Table 2.

### 3.3. Disease and Peri-Operative Details

Data regarding the timing of TAAD were available in 16/20 studies [26,27,28,31,33,34,36,37,38,39,40,41,42,43,44,45]. In total, 72.00% (95% CI, 52.45–88.64%) of our cohort suffered from acute TAAD (<14 days), 4.12% (95% CI, 0.00–19.19%) had subacute TAAD (15–90 days), while chronic dissection (>90 days) was present in 12.75% (95% CI, 2.24–47.09%) of the reported patients. The remaining 11.13% refers to the four studies that did not provide data regarding TAAD timing. Overall, 17.57% (95% CI, 0.00–65.35%) had aortic regurgitation (AR), while 15.77% (95% CI, 0.77–39.16%) suffered from organ malperfusion prior to the operation.

Vascular access through the femoral artery was preferred in 15 out of 16 included studies that reported relevant data. In detail, femoral access was chosen in 126 (57.01%) patients, femoral with adjunctive left (LCA) or right carotid artery (RCA) in 58 (26.24%), femoral with adjunctive LCA in 24 (10.85%), femoral with adjunctive brachial artery in 9 (4.07%), LCA in 2 (0.90%), and the transapical approach was chosen in 2 (0.90%) patients.

To avoid hemodynamic displacement forces, 84 (39.81%) patients underwent drug-induced hypotension, rapid ventricular pacing was used in 49 (23.22%) patients, while the inferior vena cava was transiently obstructed in 16 (7.58%) patients. Lastly, hypotension through ventricular tachycardia was induced in four (1.89%) patients by touching the left ventricle with the nose of the cone of the delivery system. Details regarding the devices used, vascular access, and cardiac output suppression mechanisms are provided in Table 3.

Furthermore, 37 (16.06% (95% CI, 1.61–37.26%)) patients underwent concomitant procedures during TEVAR. Among them, 13 (35.13%) underwent LCA-RCA bypass, 11 (29.72%) had LCA-Left subclavian artery (LSA) bypass, 3 (8.10%) had an aorta-LSA bypass, 2 had a transcatheter aortic valve replacement (TAVR), 1 (2.70%) had an aorta-LCA bypass and another patient (2.70%) underwent innominate artery scalloping. Mean operative time was 169.40 ± 30.70 min. Mean length of stay in the intensive care unit was 2.5 ± 0.9 days. The average length of hospital stay was 11.4 ± 4.2 days. Only 0.64% (95% CI, 0.00–5.34%) of the cases were converted to open salvage surgery.

### 3.4. Complications

The thirty-day complication rate was 7.08% (95% CI, 1.52–14.97%) (n = 33 patients), while 58 patients. (16.89% (95% CI, 7.75–27.88%)) developed late complications (>30 days after the operations). Overall, 8.52% of the participants. (95% CI, 2.15–17.24%) experienced endoleaks, 0.22% (95% CI, 0.00–2.11%) had technical failure during deployment of the graft as reported by the authors, and 0.10% (95% CI, 0.00–1.73%) experienced a post-operative stroke. Details about early and late morbidity including conversion to open surgery are provided in Table 4.

### 3.5. Reinterventions

**Figure 1:**
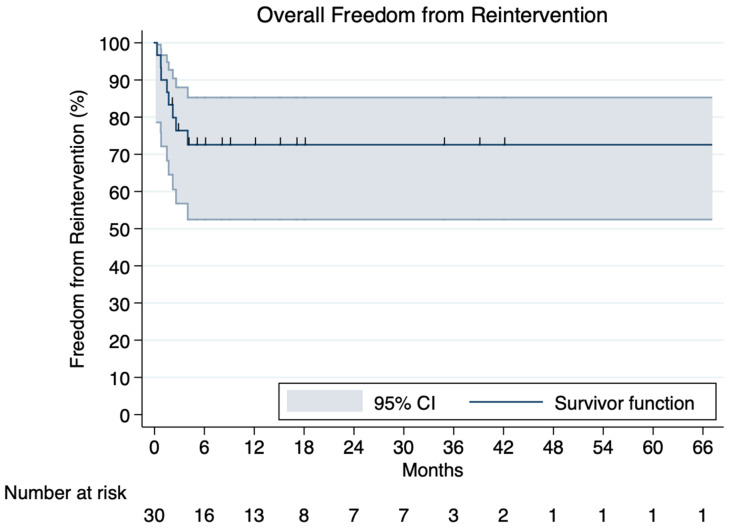
Kaplan–Meier curve demonstrating overall freedom from reintervention constructed by individualized patient data.

Reinterventions were required in 8.38% (95% CI, 1.68–17.90%) (n = 36) of the patients within a mean follow-up time of 32.40 ± 24.40 months. In total, 12 (33.33%) required an open repair, 6 (16.67%) underwent additional TEVAR, 4 (11.11%) had an aortic cuff installed, 4 (11.11%) underwent balloon expansion, 3 (8.33%) had embolization, 1 (2.77%) had an axillo-axillary bypass, and 1 (2.77%) had a TAVR. Peri-operative findings are summarized in Table 5 and Table 6 and a reconstructed Kaplan–Meier curve regarding freedom from reintervention is demonstrated in Figure 1. A diagram regarding reintervention rates is demonstrated in Supplementary Figure S2.

### 3.6. Overall Survival and Mortality

Pooled 30-day and late mortality rates were 2.46% (95% CI, 0.31–5.90%) and 1.59% (0.00–6.84%), respectively. Forest plots demonstrating pooled event rates for mortality are provided in Supplementary Figures S3 and S4.

**Figure 2:**
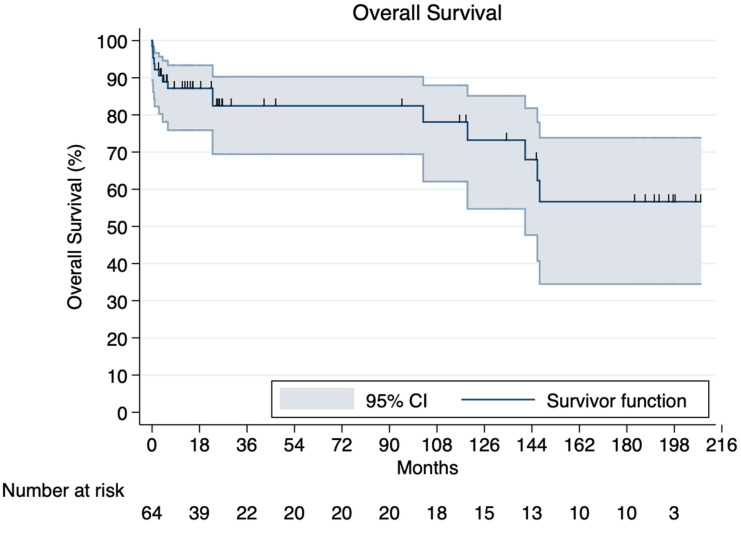
Kaplan–Meier curve demonstrating overall survival constructed by individualized patient data.

Based on the IPD, one-, three-, and five-year survival rates following TEVAR were 87.15%, 82.52% and 82.31%, respectively (Figure 2). Side-by-side comparison of the original KM curves and our reconstructed ones are provided in Supplementary Figure S5.

## 4. Discussion

The American Heart Association and the American College of Cardiology jointly updated aortic disease treatment guidelines in 2022. In this statement, endovascular treatment of TAAD was not formally endorsed due to scarcity of high-quality cumulative data [8]. The present systematic review addresses this gap in the literature and constitutes the most comprehensive synopsis of TAAD management with TEVAR to date [26,27,28,29,30,31,32,33,34,35,36,37,38,39,40,41,42,43,44,45]. Herein, mean patient age at the time of intervention was 60.7 years. The main reasons that TEVAR was preferred over open surgery included advanced age, a history of prior cardiac surgery, and serious comorbidities (i.e., severe COPD, a history of stroke, end stage renal failure, etc.) [46]. Although likely that all patients underwent endovascular treatment for similar reasons, the incentive for choosing TEVAR over open surgery was not reported in approximately half of the studies.

In our analysis, the technical failure rate was 0.22% (95% CI, 0.00–2.11%), which is substantially lower compared to historical data (8–23%) [10]. This discrepancy may be at least partly attributed to differences in the definition of TEVAR success. For example, Tsilimparis et al. recognized technical success as the uneventful deployment of the graft at the targeted landing zone [44]. Vallabhajosyula et al. defined technical success as device deployment with no intraoperative endoleak [41]. Ronchey et al. [27] and Ye et al. [39] determined technical success as the complete exclusion of the TAAD entry tear.

The overall endoleak rate in our pooled analysis was less than 9% which is approximately two times lower compared to both preliminary reports of ascending aortic TEVAR (16%) and endovascular approaches for descending aortic disease (18%) [47]. Not surprisingly, most endoleaks following TEVAR for TAAD were type I. Our pooled analysis revealed an early mortality rate of 2.46% (95% CI, 0.31–5.90%) in patients undergoing endovascular treatment of TAAD. On IPD-level analysis, one-, three-, and five-year survival rates following TEVAR were 87.15%, 82.52% and 82.31%, respectively. In highly selected patients with favorable aortic anatomy, survival with endovascular treatment appears to be superior to medical management alone (which carries a mortality rate of up to 60%) [9,48]. Interestingly enough, prognosis in our series was also substantially improved compared to previous meta-analyses where the overall mortality rate was reported to be as high as 17% (95% CI, 10–26%) [10]. Occlusion of the innominate artery as well as wire and device manipulation within the ascending aorta is thought to predispose to cerebrovascular accidents. These concerns were not validated herein since strokes developed in only 0.1% of our series. In the present study, pooled reintervention rates were 8.38% (95% CI, 1.68–17.90%) over a mean follow-up of 32.4 months. Similarly, these metrics appear to be 50% lower than previously reported (18%) [10].

In our study, 0.64% (95% CI, 0.00–5.34%) of the patients required conversions to open surgery. That said, we highly recommend keeping perfusion on standby in case conversion is warranted. Historically, the first successful endovascular repair of TAAD was reported by Dorros et al. in 2000 [49]. Even though there is no universally accepted operative technique, most centers favor the femoral artery for vascular access. Exceptions were noted by Wang et al. [30], Ye et al. [39], and Vallabhajosyula et al. [41], who utilized the left brachial, left common carotid, and left subclavian arteries, respectively. Shah et al. [50], also described the transapical method as an alternative for patients with unfavorable femoral anatomy.

Various descending aortic stent grafts have been deployed in the ascending aorta (Talent or VALOR graft (Medtronic, Santa Rosa, CA, USA), W. L. Gore & Associates (Flagstaff, Arizona), Jotec (Hechingen, Germany), Zenith TX2 (Cook Medical, Ind., Bloomington, IN, USA)) [48]. Only the 65-mm Zenith Ascend TAA (Cook) was specifically designed for use in the ascending aorta [44]. Unique features of the ascending aorta include strong pulsatility, location-dependent hemodynamic shear stress forces, and finite length. As a result, grafts shorter than 10 cm are appropriate for ascending aortic TEVAR. Delivery systems also need to account for curved anatomy whilst accommodating crossing the aortic valve (if need be) [47]. Even though no cases of retrograde dissection from the entry site were noted in the published literature, these events can certainly also occur.

It should also be emphasized that not every TAAD case is amenable to endovascular treatment. First, the intimal tear has to be at least 10 mm above the sinotubular junction and more than 5 mm proximal to the innominate artery [50]. Coronary artery height should also be reviewed [10]. Furthermore, the long nose cone of the delivery system needs to be threaded via the aortic valve into the left ventricle to achieve the appropriate proximal stent graft deployment. Therefore, TEVAR may not be feasible in the setting of heavily calcified aortic stenosis or in the presence of mechanical prosthesis. Endovascular therapy should also be avoided in patients with connective tissue disorders and/or annuloaortic ectasia [50]. Lastly, TEVAR is not an appropriate option in the context of acute aortic regurgitation, where at a minimum, meticulous inspection of the aortic valve is requisite, if not its repair or replacement.

Methodological strengths of the present paper include: (1) a comprehensive search of the literature using rigorous and systematic methodology, (2) detailed patient-level extraction of time-to-event data, (3) standardized quality assessment of eligible studies, and (4) construction of Kaplan–Meier statistics using IPD. The main differences between our study and the older meta-analysis by DeFreitas et al. [10] are (a) the addition of 231 more patients and (b) the reconstruction of IPD using Kaplan–Meier curves. Regarding differences in inclusion criteria, we also chose to include (1) patients with chronic TAAD (operated >90 days after diagnosis), (2) patients who underwent in situ laser fenestration-assisted TEVAR, (3) patients with organ malperfusion at the time of the operation, (4) patients who underwent TEVAR with a pre-fenestrated graft and (5) patients with the dissection entry tear located in the descending thoracic aorta.

Finally, there are several limitations in this study that should be acknowledged. To begin with, as with any systematic review, the present analysis is subject to a certain degree of selection bias. Certain studies did not report full data for every outcome of interest and all analyses were performed using publicly available information. Of note, the exact anatomic location of the primary tear in relation to aortic arch vessels was not provided in the vast majority of the studies. Nevertheless, tear location can influence the choice of treatment methods rendering more complex interventions necessary (i.e., chimney technique and simple-branch/fenestrated stent-graft repair, or double fenestrated TEVAR). Regarding the entry tear location, we chose to include all the studies that reported on patients with TAAD who were treated endovascularly. That said, in our effort to best represent the nosological entity with this specific treatment, we have included both patients with an entry tear in the ascending and the descending aorta, as long as the dissection was specified as Type A. Furthermore, nearly all included studies are observational studies with lack of randomization. Also, since we have used the pool estimation of proportion to report the outcomes, it should be highlighted that there might be significant heterogeneity and bias, which could have significantly affected our outcomes. Additionally, TEVAR procedures were performed in different medical centers by various, yet extremely experienced endovascular operators. This heterogeneity, along with variations in follow-up routines, may affect the generalizability and applicability of our results to less specialized setups. It should also be emphasized that mean follow-up was 32.40 ± 24.40 months and as such the long-term outcomes of this approach cannot be fully quantified with the present analysis. Lastly, eligible studies did not provide granular data regarding their open surgical cohorts. Therefore, we could not perform a direct comparison of surgery versus TEVAR. The scarcity of detailed timing segmentation within the existing literature constrained our ability to stratify outcomes by TAAD category (acute/subacute/chronic). Our objective was to compile and present all available data in a manner that not only adheres closely to the reality presented within the existing studies but also offers reliability and utility for aortic guidelines and providers seeking actionable information. We believe that despite the aforementioned limitations, our method of analysis holds value in that it strives to encapsulate the most accurate representation of the available data, thereby providing insights that are both relevant and applicable in a practical context.

## 5. Conclusions

The present study utilized robust patient-level meta-analysis techniques to summarize contemporary experience with endovascular treatment of type A aortic dissections. Technical failure, stroke, and conversion to open surgery occurred in less than 0.7% of the cohort. The incidence of endoleak was also remarkably low (8.52%). One-, three-, and five-year survival rates were estimated at 87.15%, 82.52% and 82.31%, respectively. Reintervention was required in less than 9% of the patients over a mean follow-up of 32.4 months. These findings suggest that TEVAR may be a viable option for poor surgical candidates with TAAD in the setting of favorable aortic anatomy and at the hands of highly experienced endovascular teams. Additional research with longer follow-up is warranted to further define the role of endovascular therapy in Type A aortic dissections.

## Figures and Tables

**Table 1:** Study characteristics of the included manuscripts.

Author	Year	Journal	Center	Country/Region	Design	Study Period	TEVAR
Hsieh et al. 2019 [26]	2019	*Journal of Cardiac Surgery*	Changhua Christian Hospital	Taiwan	Retrospective cohort	2015–2018	4
Ronchey et al. 2013 [27]	2013	*European Journal of Vascular and Endovascular Surgery*	San Filippo Neri Hospital, Rome	Italy	Retrospective cohort	2009–2012	4
Chen et al. 2019 [28]	2019	*Annals of Vascular Surgery*	Kaohsiung Chang Gung Memorial Hospital	Taiwan	Retrospective cohort	2017–2019	4
Gao et al. 2017 [29]	2017	*Journal of Endovascular Therapy*	Jiangya Haikou Hospital, Municipal Hospital of XinJiang, XinJiang	China	Retrospective cohort	2013–2016	7
Wang et al. 2021 [30]	2021	*Vascular*	General Hospital of Ningxia Medical University, Ningxia	China	Retrospective cohort	April 2016–June 2017	9
Mitreski et al. 2022 [31]	2022	*CVIR Endovascular*	Austin Health, Heidelberg	Australia	Retrospective cohort	2011–2020	10
Roselli et al. 2018 [32]	2018	*The Journal of Thoracic and Cardiovascular Surgery*	Cleveland Clinic, Cleveland	USA	Retrospective cohort	2006–2016	14
Li et al. 2016 [33]	2016	*Journal of the American College of Cardiology*	2nd Military Medical University, Shanghai	China	Retrospective cohort	2009–2011	15
Shu et al. 2012 [34]	2012	*Journal of Vascular and Interventional Radiology*	The 2nd Xiang-ya Hospital of Central-south University, Changsha	China	Retrospective cohort	2006–2011	17
Yuan et al. 2022 [35]	2022	*Indian Journal of Thoracic and Cardiovascular Surgery*	Cardiology and Aortic Centre, Royal Brompton and Harefield Hospitals, London	UK	Retrospective cohort	2015–2020	19
Yan et al. 2019 [36]	2019	*Journal of Vascular Surgery*	Shanghai Ninth People’s Hospital, Shanghai	China	Retrospective cohort	March 2016–December 2018	20
Wu et al. 2021 [37]	2021	*Journal of Interventional Cardiology*	Union Hospital, Fujian Medical University, Fuzhou	China	Retrospective cohort	January 2018–December 2019	24
Higashigawa et al. 2019 [38]	2019	*Journal of Vascular and Interventional Radiology*	Multicenter (Japan)	Japan	Retrospective cohort	May 1997–January 2016	31
Ye et al. 2011 [39]	2011	*European Journal of Vascular and Endovascular Surgery*	The First Affiliated Hospital, Sun Yat-Sen University, Guangzhou	China	Retrospective cohort	2001–2009	45
Qin et al. 2019 [40]	2019	*EuroIntervention*	Shanghai Jiao Tong University, Shanghai	China	Retrospective cohort	April 2014–May 2018	58
Vallabhajosyula et al. 2015 [41]	2015	*The Journal of Thoracic and Cardiovascular Surgery*	University of Pennsylvania Medical Center, Philadelphia	USA	Retrospective cohort	2007–2012	2
Khoynezhad et al. 2016 [42]	2016	*Journal of Vascular Surgery*	Cedars-Sinai Medical Center, David Geffen UCLA School of Medicine, Los Angeles	USA	RCT	2014–2016	2
Bernardes at al. 2014 [43]	2014	*The Journal of Thoracic and Cardiovascular Surgery*	Madre Teresa Hospital, Belo Horizonte, Minas Gerais	Brazil	Retrospective cohort	2007–2012	2
Tsilimparis et al. 2019 [44]	2019	*Journal of Endovascular Therapy*	German Aortic Center, University Heart Center, Hamburg	Germany	Retrospective cohort	2010–2017	16
Ghoreishi et al. 2019 [45]	2019	*The Annals of Thoracic Surgery*	University of Maryland School of Medicine, Baltimore	USA	Retrospective cohort	2018–2019	8

Abbreviations: USA = United States of America; UK = United Kingdom.

**Table 2:** Details of patient demographic characteristics and medical history.

Author	TEVAR	Age (SD) (Years)	Males (n, %)	Females (n, %)	DM (n, %)	COPD (n, %)	HTN (n, %)
Hsieh et al. 2019 [26]	4	56.25 (14.4)	2 (50)	2 (50)	NR	NR	NR
Ronchey et al. 2013 [27]	4	70 (7.65)	2 (50)	2 (50)	1 (25)	NR	4 (100)
Chen et al. 2019 [28]	4	67.25 (8.5)	3 (75)	1 (25)	NR	NR	NR
Gao et al. 2017 [29]	7	54.57 (10.25)	7 (100)	0 (0)	NR	NR	NR
Wang et al. 2021 [30]	9	NR	7 (77.8)	2 (22.2)	NR	NR	8 (88.9)
Mitreski et al. 2022 [31]	10	60.7 (11.47)	6 (60)	4 (40)	NR	NR	NR
Roselli et al. 2018 [32]	14	NE	4 (28.6)	10 (71.4)	5 (35.7)	4 (28.6)	13 (92.9)
Li et al. 2016 [33]	15	65 (12.1)	12 (80)	3 (20(	NR	3 (20)	NR
Shu et al. 2012 [34]	17	54.5 (10.3)	16 (94.1)	1 (5.9)	4 (23.5)	0 (0)	17 (100)
Yuan et al. 2022 [35]	19	NR	NR	NR	NR	NR	NR
Yan et al. 2019 [36]	20	67 (NR)	18 (90)	2 (10)	9 (45)	6 (30)	13 (65)
Wu et al. 2021 [37]	24	65.4 (9.3)	22 (91.7)	2 (8.3)	3 (12.5)	2 (8.3)	23 (95.8)
Higashigawa et al. 2019 [38]	31	64 (11.0)	30 (96.8)	1 (3.2)	1 (3.2)	1 (3.2)	22 (70.9)
Ye et al. 2011 [39]	45	51 (NR)	41 (91.1)	4 (8.9)	NR	NR	41 (91.1)
Qin et al. 2019 [40]	58	58 (NR)	32 (55.1)	26 (44.9)	33 (56.9)	4 (6.9)	42 (72.4)
Vallabhajosyula et al. 2015 [41]	2	84 (NR)	0 (0)	2 (100)	0 (0)	0 (0)	2 100)
Khoynezhad et al. 2016 [42]	2	86 (NR)	1 (50)	1 (50)	0 (0)	NR	1 (50)
Bernardes at al. 2014 [43]	2	52.5 (NR)	0 (0)	2 (100)	0 (0)	0 (0)	2 (100)
Tsilimparis et al. 2019 [44]	16	70 (15)	NE	NE	NR	NR	NR
Ghoreishi et al. 2019 [45]	8	69 (9)	NE	NE	NR	1 (12.5)	8 (100)

NR = not reported; NE = non-extractable; DM = Diabetes Mellitus; COPD = chronic obstructive pulmonary disease; HTN = hypertension.

**Table 3:** Vascular access, devices, and cardiac output suppression mechanisms.

Author	TEVAR	Access Site (n, %)	Device Used (n, %)	Mechanism of Cardiac Output Suppression (n, %)
Hsieh et al. 2019 [26]	4	Femoral (n = 4, 100)	GORE (n = 2, 50), MEDTRONIC (n = 1, 25)	VTACH (n = 4, 100)
Ronchey et al. 2013 [27]	4	Femoral (n = 4, 100)	Cook TX2 (n = 1, 25), Off-shelf Cook (n = 3, 75)	RVP (n = 4, 100)
Chen et al. 2019 [28]	4	Femoral (n = 4, 100)	NR	DHTN (n = 4, 100)
Gao et al. 2017 [29]	7	Femoral (n = 7, 100)	GORE TAG (n = 7, 100)	DHTN (n = 7, 100)
Wang et al. 2021 [30]	9	Femoral with adjunctive brachial (n = 9, 100)	MEDTRONIC Valiant Captiva (n = 9, 100)	DHTN (n = 9, 100%
Mitreski et al. 2022 [31]	10	Femoral (n = 10, 100)	COOK Zenith TX2 (n = 10, 100)	NR
Roselli et al. 2018 [32]	14	NR	NR	RVP (n = 14, 100)
Li et al. 2016 [33]	15	Femoral (n = 15, 100)	COOK Zenith TX2 Pro Form (n = 15, 100)	NR
Shu et al. 2012 [34]	17	Femoral (n = 17, 100)	MicroPort Hercules (n = 10, 58.9), COOK Zenith (n = 5, 29.4), MEDTRONIC Valiant (n = 2, 11.8)	DHTN (n = 17, 100
Yuan et al. 2022 [35]	19	Femoral (n = 19, 100)	NR	RVP (n = 19, 100)
Yan et al. 2019 [36]	20	Femoral (n = 20, 100)	GORE TAG (n = 20, 100)	NR
Wu et al. 2021 [37]	24	Femoral with adjunctive LCA (n = 24, 100)	NR	NR
Higashigawa et al. 2019 [38]	31	NR	NR	NR
Ye et al. 2011 [39]	45	LCA (n = 2, 4.4), femoral (n = 22, 48.8)	NR	DHTN (n = 45, 100)
Qin et al. 2019 [40]	58	Femoral with adjunctive LCA or RCA (n = 58, 100)	GORE TAG (n = 58, 100)	SCA (n = 58, 100)
Vallabhajosyula et al. 2015 [41]	2	Transapical (n = 2, 100)	COOK Zenith TX2 (n = 2, 100)	RVP (n = 2, 100)
Khoynezhad et al. 2016 [42]	2	Femoral (n = 2, 100)	MEDTRONIC Valiant PS-IDE (n = 2, 100)	RVP (n = 2, 100)
Bernardes at al. 2014 [43]	2	Femoral (n = 2, 100)	COOK Zenith (n = 1, 50), GORE TAG (n = 1, 50)	DHTN (n = 2, 100)
Tsilimparis et al. 2019 [44]	16	NR	Cook Ascend TAA Endovascular Graft (n = 16, 100)	IVCO (n = 16, 100)
Ghoreishi et al. 2019 [45]	8	NR	GORE TAG (n = 8, 100)	RVP (n = 8, 100)

NR = not reported; VTACH = ventricular tachycardia; RVP = rapid ventricular pacing; SCA = systemic circulatory arrest; IVCO = inferior vena cava occlusion; DHTN = drug-induced hypotension.

**Table 4:** Cumulative data about complications and conversion to open surgery rates.

Author	TEVAR	Early Complications (n, %)	Early Complications (Types) (n, %)	Late Complications (n, %)	Late Complications (Types) (n, %)	Conversion to Open Surgery (n, %)
Hsieh et al. 2019 [26]	4	1 (25)	New TAAD (n = 1, 100)	2 (50)	Endoleak (n = 1, 50)	1 (25)
Ronchey et al. 2013 [27]					New TAAD (n = 1, 50)	
Chen et al. 2019 [28]	4	0 (0)	0 (0)	0 (0)	0 (0)	0 (0)
Gao et al. 2017 [29]	4	0 (0)	0 (0)	3 (75)	New PAU (n = 1, 33.3)	0 (0)
Wang et al. 2021 [30]					Hematoma progression (n = 1, 33.3)	
Mitreski et al. 2022 [31]					New TAAD (n = 1, 33.3)	
Roselli et al. 2018 [32]	7	0 (0)	0 (0)	0 (0)	0 (0)	0 (0)
Li et al. 2016 [33]	9	0 (0)	0 (0)	2 (22.2)	New TAAD (n = 1, 50)	0 (0)
Shu et al. 2012 [34]					Endoleak (n = 1, 50)	
Yuan et al. 2022 [35]	10	2 (20)	Pulmonary hemorrhage (n = 1, 50)	0 (0)	0 (0)	NR
Yan et al. 2019 [36]			End-organ ischemia (n = 1, 50)			
Wu et al. 2021 [37]	14	6 (42.8)	Cardiac tamponade (n = 2, 33.3)	4 (28.6)	NR	NR
Higashigawa et al. 2019 [38]			Multi-organ-failure (n = 2, 33.3)			
Ye et al. 2011 [39]	15	2 (13.3)	Arrhythmia (n = 1, 50)	8 (53.3)	New TAAD (n = 2, 25)	1 (6.7)
Qin et al. 2019 [40]					Myocardial Infarction (1, 12.5)	
Vallabhajosyula et al. 2015 [41]					Arrhythmia (1, 12.5)	
Khoynezhad et al. 2016 [42]					Tamponade (1, 12.5)	
Bernardes at al. 2014 [43]					Endoleak (n = 1, 12.5)	
Tsilimparis et al. 2019 [44]	17	0 (0)	0 (0)	1 (5.9)	New TAAD (n = 1, 100)	0 (0)
Ghoreishi et al. 2019 [45]	19	0 (0)	0 (0)	1 (5.3)	Endoleak (n = 1, 100)	1 (5.3)
Hsieh et al. 2019 [26]	20	2 (10)	Pneumonia (n = 1, 50)	3 (15)	Endoleak (n = 3, 100)	0 (0)
Ronchey et al. 2013 [27]			Stroke (n = 1, 50)			
Chen et al. 2019 [28]	24	0 (0)	0 (0)	1 (4.2)	Endoleak (n = 1, 100)	0 (0)
Gao et al. 2017 [29]	31	8 (25.8)	Abdominal aortic rupture (n = 1, 12.5)	9 (29.0)	Intimal injury (n = 3, 33.3)	5 (16.1)
Wang et al. 2021 [30]			Intimal injury (n = 3, 37.5)		Abdominal aortic aneurysm (n = 1, 11.1)	
Mitreski et al. 2022 [31]			Left arm ischemia (n = 1, 12.5)		New TAAD (n = 3, 33.3)	
Roselli et al. 2018 [32]			Type I endoleak (n = 2, 25)		Type I endoleak (n = 1, 11.1)	
Li et al. 2016 [33]			Type II endoleak (n = 1, 12.5)		Type II endoleak (n = 1, 11.1)	
Shu et al. 2012 [34]	45	4 (8.9)	Myocardial infarction (n = 1, 25)	14 (31.1)	Type I endoleak (n = 9, 75.4)	0 (0)
Yuan et al. 2022 [35]			Neck hematoma (n = 1, 25)		Stroke (n = 3, 21.4)	
Yan et al. 2019 [36]			Gastrointestinal hemorrhage (n = 1, 25)		Pseudoaneurysm (n = 1, 7.1)	
Wu et al. 2021 [37]			Type I endoleak (n = 1, 25)		Type II endoleak (n = 1, 7.1)	
Higashigawa et al. 2019 [38]	58	2 (3.4)	Cardiac tamponade (n = 1, 50)	3 (5.2)	Type I endoleak (n = 2, 66.7)	0 (0)
Ye et al. 2011 [39]			Pneumonia (n = 1, 50)		Type II endoleak (n = 1, 33.3)	
Qin et al. 2019 [40]	2	1 (50)	Aortic valve leaflet entrapment (n = 1, 100)	1 (50)	Endoleak (n = 1, 100)	0 (0)
Vallabhajosyula et al. 2015 [41]	2	1 (50)	NR	1 (50)	New PAU (n = 1, 100)	1 (50)
Khoynezhad et al. 2016 [42]	2	1 (50)	Endoleak (n = 1, 100)	2 (100)	Pneumonia (n = 1, 50)	2 (100)
Bernardes at al. 2014 [43]					Pulmonary Embolism (n = 1, 50)	
Tsilimparis et al. 2019 [44]	16	NR	NR	NE	NE	NR
Ghoreishi et al. 2019 [45]	8	3 (37.5)	Cardiac tamponade (n = 1, 33.3)	1 (12.5)	Aortic regurgitation (n = 1, 100)	0 (0)
			Multi-organ failure (n = 1, 33.3)			
			Right common femoral artery dissection (n = 1, 33.3)			

Abbreviations: TAAD = Type A aortic dissection; TEVAR = thoracic endovascular aortic repair; PAU = penetrating aortic ulcer; NE = non-extractable data; NR = not reported.

**Table 5:** Cumulative disease and peri-operative details.

Author	TEVAR	Acute TAAD (n, %)	Subacute TAAD (n, %)	Chronic TAAD (n, %)	Operation Time (SD) (Minutes)	Follow-Up Time (SD) (Months)	Concomitant Procedures (n)	Mean Hospital Stay (SD) (Days)	Mean ICU Stay (SD) (Days)
Hsieh et al. 2019 [26]	4	4 (100)	0 (0)	0 (0)	NR	11 (NR)	1	NR	NR
Ronchey et al. 2013 [27]	4	4 (100)	0 (0)	0 (0)	128 (NR)	15 (NR)	1	NR	NR
Chen et al. 2019 [28]	4	4 (100)	0 (0)	0 (0)	NR	12.25 (5.1)	0	NR	NR
Gao et al. 2017 [29]	7	NR	NR	NR	231.6 (38.4)	14.3 (13.4)	4	NR	NR
Wang et al. 2021 [30]	9	NR	NR	NR	152.22 (17.81)	46.5 (4.47)	NR	NR	NR
Mitreski et al. 2022 [31]	10	9 (90)	0 (0)	1	NR	36.6 (NR)	0	NR	NR
Roselli et al. 2018 [32]	14	NR	NR	NR	NR	NR	0	NR	NR
Li et al. 2016 [33]	15	1 (6.7)	7 (46.7)	7 (46.7)	128.6 (26.2)	62 (NR)	NR	9.4 (2.5)	3.3 (1)
Shu et al. 2012 [34]	17	2 (11.8)	0 (0)	15 (88.2)	82.1 (16.9)	25.7 (17.2)	NR	NR	NR
Yuan et al. 2022 [35]	19	NR	NR	NR	NR	NR	NR	NR	NR
Yan et al. 2019 [36]	20	20 (100)	0 (0)	0 (0)	208 (NR)	16 (NR)	NR	13 (5)	2 (NR)
Wu et al. 2021 [37]	24	15 (62.5)	0 (0)	9 (37.5)	237.4 (40.6)	21.4 (6.9)	NR	15.5 (7.1)	1.8 (1.1)
Higashigawa et al. 2019 [38]	31	24 (77.4)	7 (29.1)	0 (0)	NR	99 (69.0)	NR	NR	NR
Ye et al. 2011 [39]	45	30 (66.7)	0 (0)	15 (33.3)	NR	35.5 (5.4)	20	NR	NR
Qin et al. 2019 [40]	58	21 (36.2)	37 (63.8)	0 (0)	162 (36)	10.6 (5.4)	NR	10 (2.6)	NR
Vallabhajosyula et al. 2015 [41]	2	2 (100)	0 (0)	0 (0)	NR	NR	0	NR	10 (NR)
Khoynezhad et al. 2016 [42]	2	1 (50)	1 (50)	0 (0)	NR	NR	1	NR	NR
Bernardes at al. 2014 [43]	2	2 (100)	0 (0)	0 (0)	NR	NR	0	NR	NR
Tsilimparis et al. 2019 [44]	16	8 (50)	0 (0)	8 (50)	NE	11 (NR)	10	NR	NR
Ghoreishi et al. 2019 [45]	8	7 (87.5)	0 (0)	1 (12.5)	NE	13 (13)	0	8.6 (NR)	NR

NR = not reported; NE = non-extractable, TAAD = Type A aortic dissection; ICU = intensive care unit.

**Table 6:** Cumulative disease and peri-operative details.

Author	Early Complications (n, %)	Late Complications (n, %)	Endoleak (n, %)	Technical Failure (n, %)	Stroke	Reintervention (n, %)	Conversion to Open Surgery (n, %)
Hsieh et al. 2019 [26]	1 (25%)	2 (50)	1 (25)	1 (25)	0 (0)	1 (25)	1 (25)
Ronchey et al. 2013 [27]	0 (0)	0 (0)	NR	0 (0)	0 (0)	0 (0)	0 (0)
Chen et al. 2019 [28]	0 (0)	3 (75)	0 (0)	0 (0)	0 (0)	2 (50)	1 (25)
Gao et al. 2017 [29]	0 (0)	0 (0)	NR	0 (0)	0 (0)	0 (0)	0 (0)
Wang et al. 2021 [30]	0 (0)	2 (22.2)	1 (11.1)	0 (0)	0 (0)	1 (11.1)	0 (0)
Mitreski et al. 2022 [31]	2 (20)	0 (0)	NR	0 (0)	0 (0)	1 (10)	NR
Roselli et al. 2018 [32]	6 (42.8)	4 (28.6)	NR	0 (0)	2 (14.7)	NR	NR
Li et al. 2016 [33]	2 (13.3)	8 (53.3)	1 (6.7)	0 (0)	0 (0)	2 (13.1)	1 (6.7)
Shu et al. 2012 [34]	0 (0)	1 (5.9)	NR	0 (0)	0 (0)	1 (5.9)	0 (0)
Yuan et al. 2022 [35]	0 (0)	1 (5.3)	NR	2 (10.5)	1 (5.3)	1 (5.3)	1 (5.3)
Yan et al. 2019 [36]	2 (10)	3 (15)	3 (15)	0 (0)	1 (5)	0 (0)	0 (0)
Wu et al. 2021 [37]	0 (0)	1 (4.2)	1 (4.2)	0 (0)	0 (0)	0 (0)	0 (0)
Higashigawa et al. 2019 [38]	8 (25.8)	9 (29.0)	5 (16.1)	2 (6.4)	0 (0)	10 (32.3)	4 (12.8)
Ye et al. 2011 [39]	4 (8.9)	14 (31.1)	11 (24.4)	1 (2.2)	3 (6.7)	10 (22.2)	0 (0)
Qin et al. 2019 [40]	2 (3.4)	3 (5.2)	3 (5.2)	5 (8.6)	2 (3.4)	0 (0)	0 (0)
Vallabhajosyula et al. 2015 [41]	1 (50)	1 (50)	2 (100)	0 (0)	1 (50)	0 (0)	0 (0)
Khoynezhad et al. 2016 [42]	1 (50)	1 (50)	0 (0)	0 (0)	0 (0)	1 (50)	1 (50)
Bernardes at al. 2014 [43]	1 (50)	2 (100)	1 (50)	0 (0)	0 (0)	2 (100)	2 (100)
Tsilimparis et al. 2019 [44]	NE	NE	NR	NR	NR	3 (18.8)	NR
Ghoreishi et al. 2019 [45]	3 (37.5)	1 (12.5)	0 (0)	NR	0 (0)	1 (12.5)	0 (0)

NR = not reported; NE = non-extractable.

## Data Availability

Data available in a publicly accessible repository. The data presented in this study are openly available in the sources provided in the references section.

## Supplementary Materials

The following supporting information can be downloaded at: https://www.mdpi.com/article/10.3390/jcm12227051/s1, Figure S1: Flow diagram in accordance with the PRISMA 2020 guidelines; Figure S2: Forest Plot diagram demonstrating data for reintervention rates; Figure S3: Forest Plot diagram demonstrating data for early mortality event rates; Figure S4: Forest Plot diagram demonstrating data for late mortality event rates; Figure S5: Provided and Reconstructed Kaplan–Meier curves for observed survival from Higashigawa et al. [38]; Figure S6: Provided and Reconstructed Kaplan–Meier curves for observed survival from Yan et al. [36]; Table S1: Risk of bias assessment using the NHLBI tool.
