# Supplementary material for: Endovascular Treatment of Type A Aortic Dissection: A Systematic Review and Meta-Analysis Using Reconstructed Time-to-Event Data
Source: J Clin Med. 2023 Nov 12;12(22):7051. doi: 10.3390/jcm12227051 (PMC10672308; doi:10.3390/jcm12227051)
Supplement: Supplementary file 1 [file jcm-12-07051-s001.zip › jcm-2685513-supplementary.pdf]

Journal of
Clinical Medicine

Supplemental Figure S1: Flow diagram in accordance with the PRISMA 2020 guidelines.

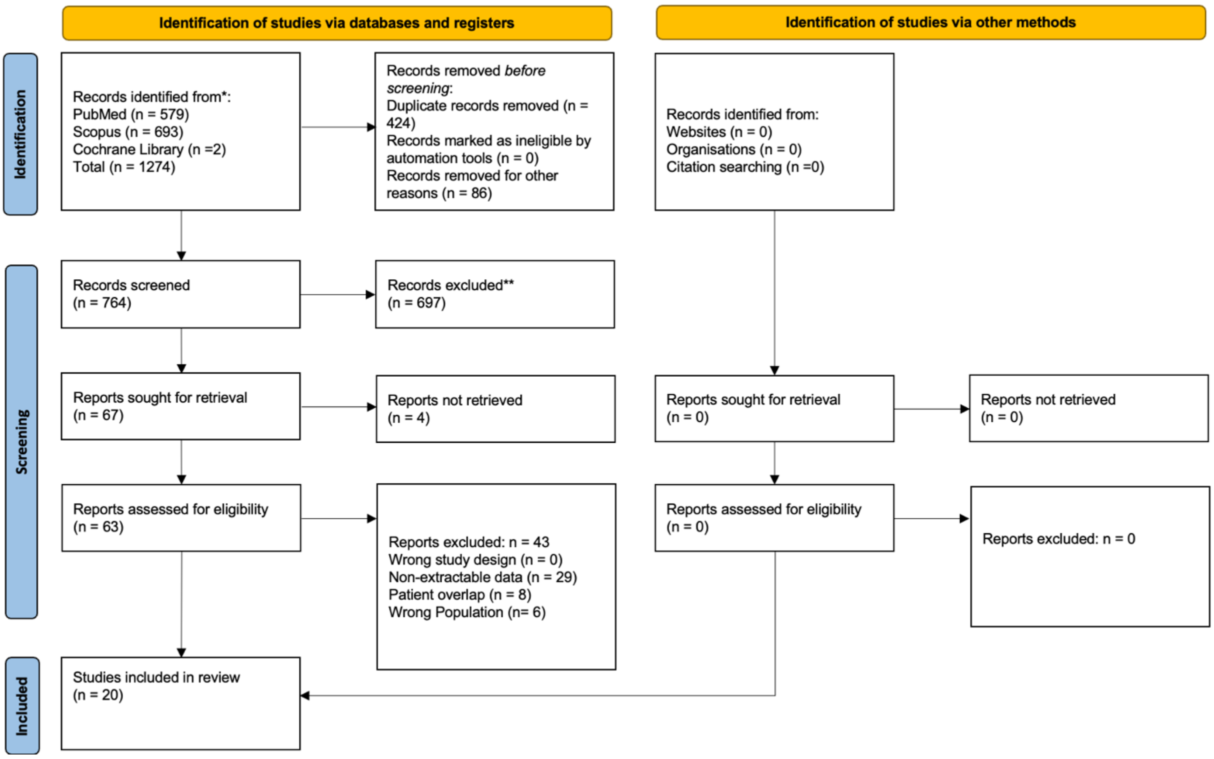

*Consider, if feasible to do so, reporting the number of records identified from each database or register searched (rather than the total number across all databases/registers).

**If automation tools were used, indicate how many records were excluded by a human and how many were excluded by automation tools.

From: Page MJ, McKenzie JE, Bossuyt PM, Boutron I, Hoffmann TC, Mulrow CD, et al. The PRISMA 2020 statement: an updated guideline for reporting systematic reviews. BMJ 2021;372:n71. doi: 10.1136/bmj.n71. For more information, visit: http://www.prisma-statement.org/

Supplemental Figure S2: Forest Plot diagram demonstrating data for reintervention rates.

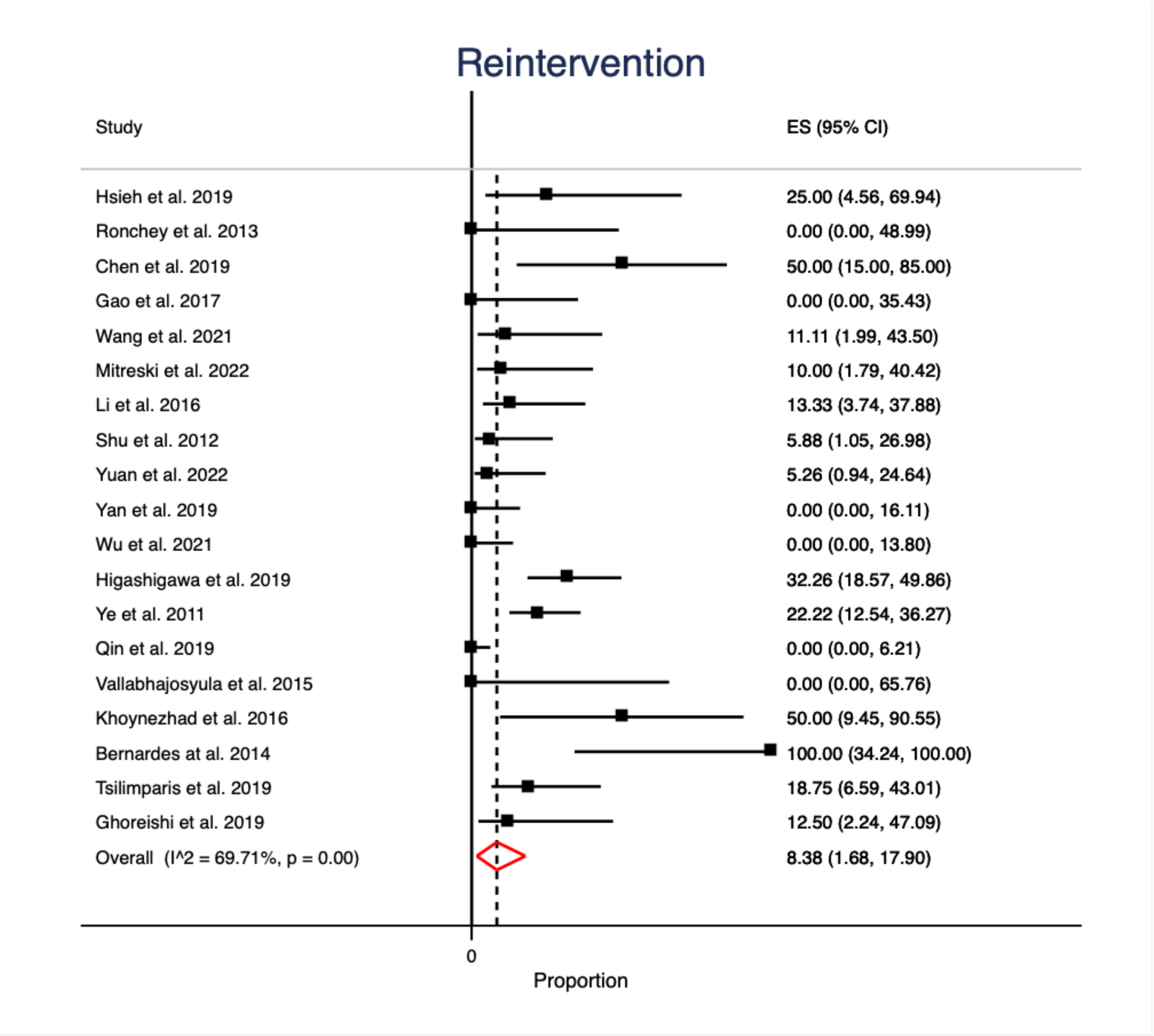

Supplemental Figure S3: Forest Plot diagram demonstrating data for early mortality event rates.

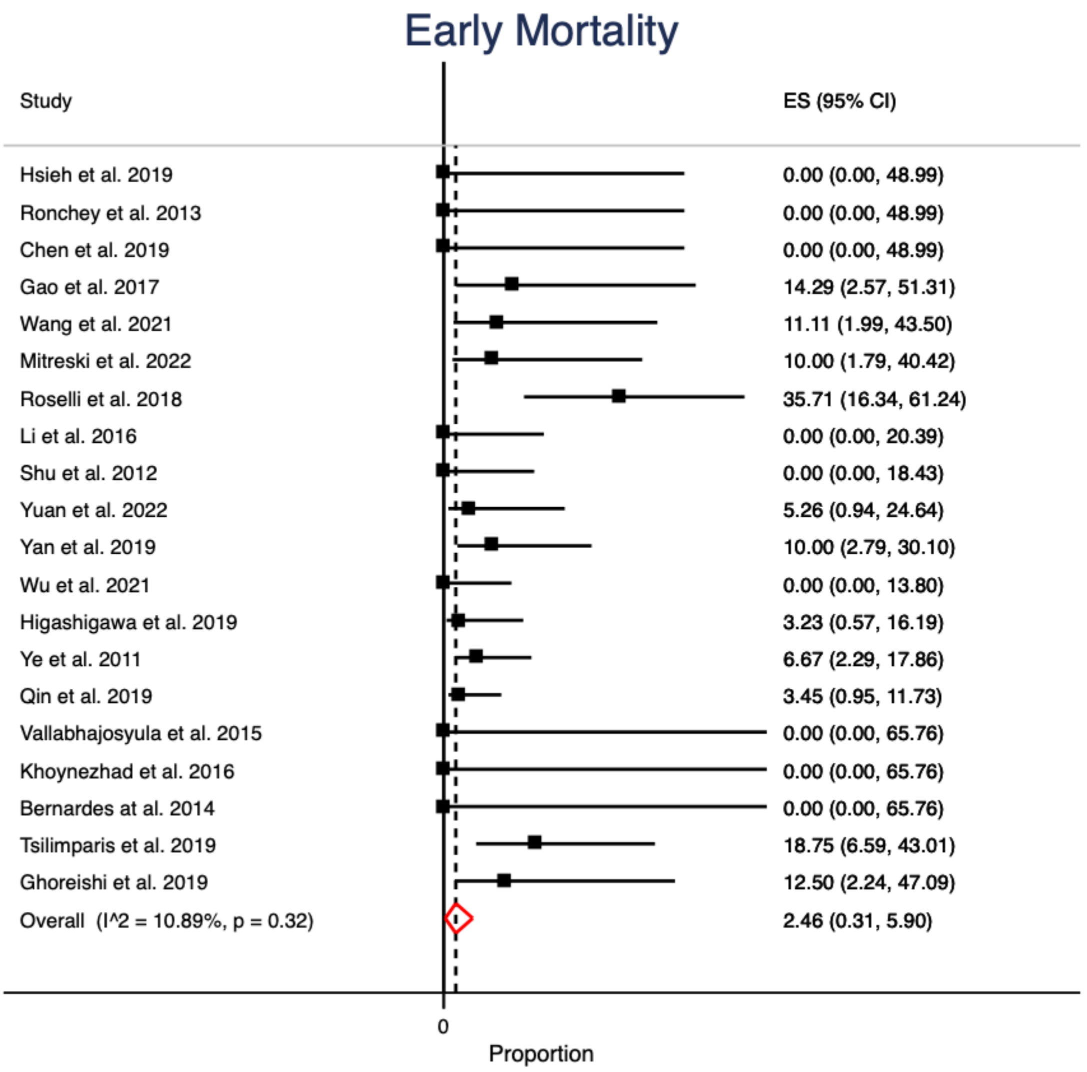

Supplemental Figure S4: Forest Plot diagram demonstrating data for late mortality event rates.

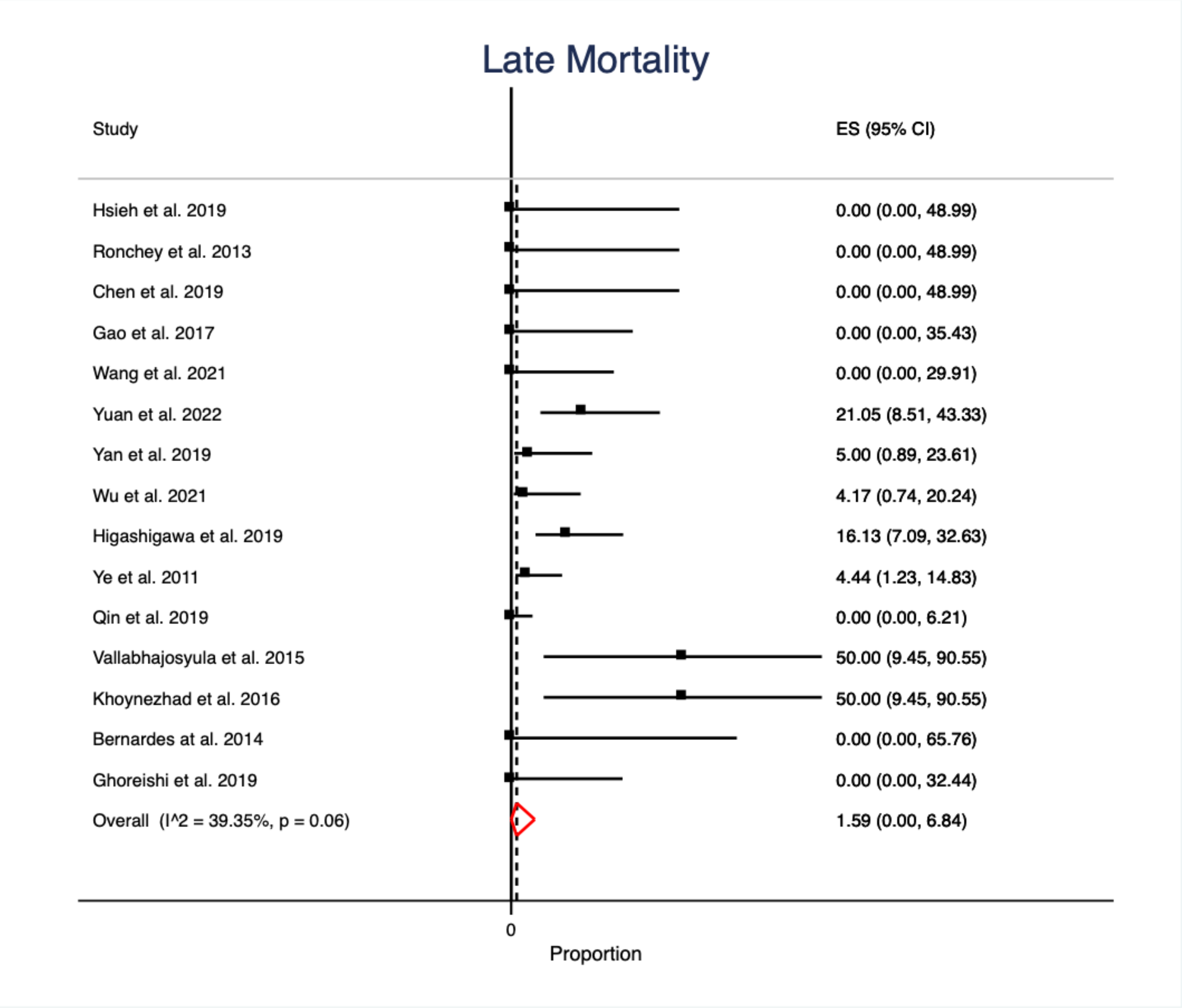

Supplemental Figure S5: Provided and Reconstructed Kaplan–Meier curves for observed survival from Higashigawa et al.

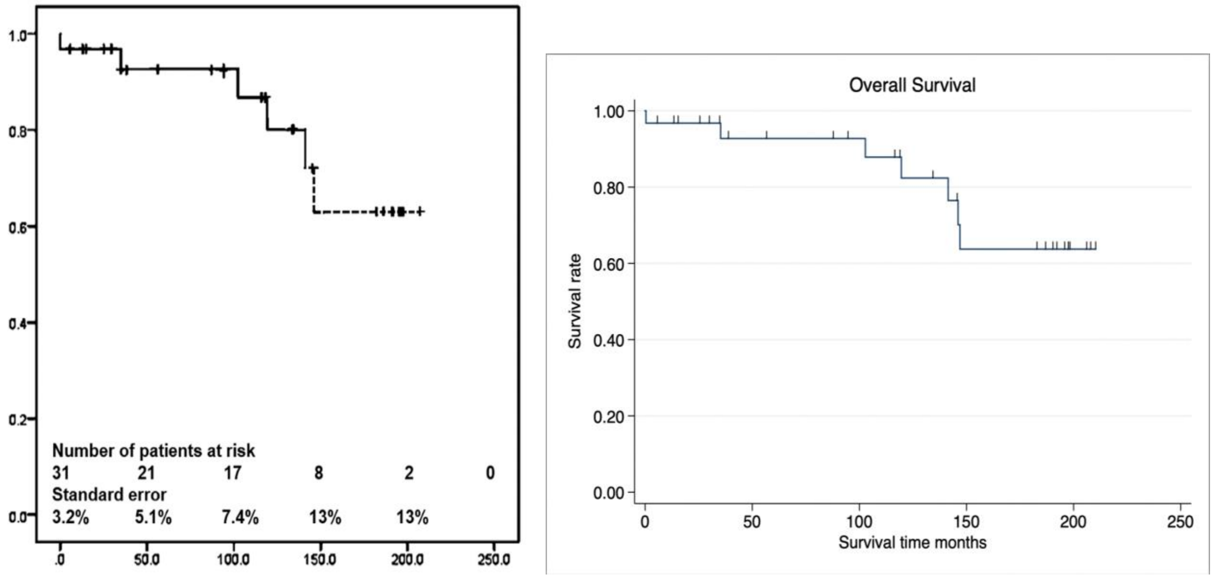

Supplemental Figure S6: Provided and Reconstructed Kaplan–Meier curves for observed survival from Yan et al.

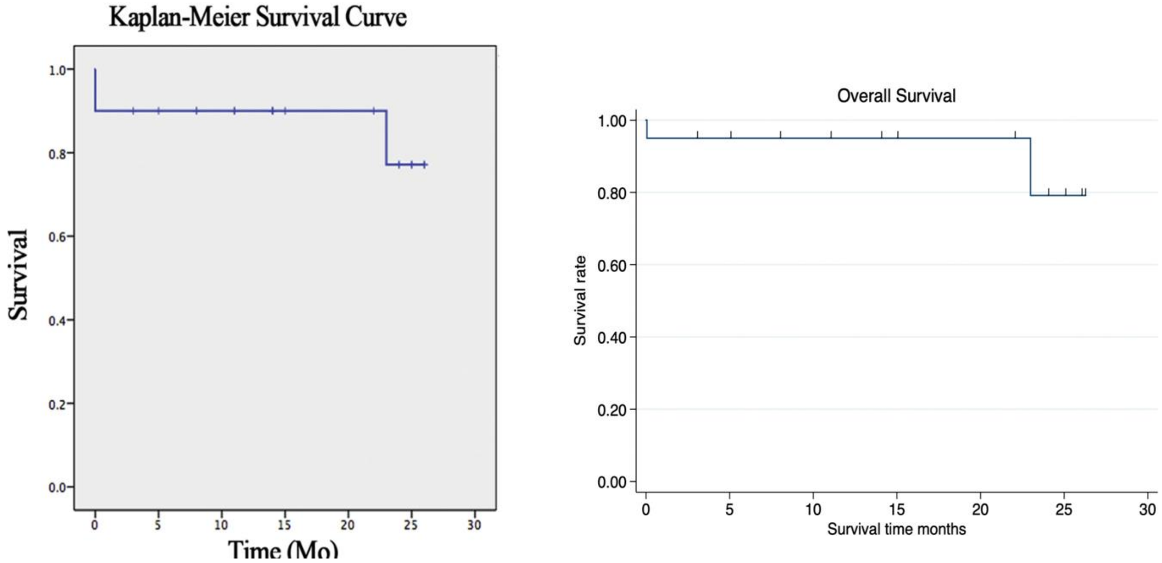

Supplemental Table S1: Risk of bias assessment using the NHLBI tool.

Author	Study question or objective clearly stated?	Study population clearly and fully described, including a case definition?	Were the cases consecutive?	Were the subjects comparable?
Hsieh et al. 2019 [29]	YES	YES	YES	YES
Ronchey et al. 2013 [30]	YES	YES	YES	YES
Chen et al. 2019 [31]	YES	YES	YES	YES
Gao et al. 2017 [42]	YES	YES	YES	YES
Wang et al. 2021 [43]	YES	YES	YES	YES
Mitreski et al. 2022 [32]	YES	YES	YES	YES
Roselli et al. 2018 [44]	YES	YES	YES	YES
Li et al. 2016 [33]	YES	YES	YES	YES
Shu et al. 2012 [34]	YES	YES	YES	YES
Yuan et al. 2022 [45]	YES	YES	YES	YES
Yan et al. 2019 [27]	YES	YES	YES	YES
Wu et al. 2021 [35]	YES	YES	YES	YES
Higashigawa et al. 2019 [37]	YES	YES	YES	YES
Ye et al. 2011 [36]	YES	YES	YES	YES
Qin et al. 2019 [26]	YES	YES	YES	YES
Vallabhajosyula et al. 2015 [28]	YES	YES	YES	YES
Khoynezhad et al. 2016 [39]	YES	YES	YES	YES
Bernardes at al. 2014 [38]	YES	YES	YES	YES
Tsilimparis et al. 2019 [40]	YES	YES	YES	YES
Ghoreishi et al. 2019 [41]	YES	YES	YES	YES
Abbreviations: NR=Not Reported				
